# Clinical and Pathological Reports of Cases of Insanity

**Published:** 1884-06

**Authors:** S. V. Clevenger

**Affiliations:** Special Pathologist Cook County Insane Asylum


### ARTICLE III.

---

### CLINICAL AND PATHOLOGICAL REPORTS OF CASES OF INSANITY. By S. V. CLEVENGER, M.D., Special Pathologist Cook County Insane Asylum.

Male case No. 317. Paretic Dementia. P. M., age 47; admitted Nov. 7, 1883; American; married; salesman. Delusions of grandeur; President of U. S., Son of God; hallucinations of sight; noisy; errabund; mother was "insane a short time," and died of heart-disease in 1878; father of "ulcer of leg." When 20 years old patient was in Indianapolis Asylum three months. He had been drinking hard previous to this. Last attack came on while hard at work; was furious; broke windows, etc. A large dose of fluid extract conium gave him a twenty-four hour sleep, which he needed badly, having been furibund night and day for some weeks. He awoke refreshed, and remained apparently in full possession of his faculties, quiet, gentlemanly, and free from delusions until the last of December, when remission gave place to excitement. The Trinity, Masonry and Politics afford him special topics. Is rapidly deteriorating. There is a characteristic proclivity in paretics to emphasize a great deal in the documents they are fond of writing. Here is a specimen: "I shall start out on a *Mission of Peace* taking for mottoes *Truth*, *Justice*, *Liberty*, and endeavor with all the *earnest vigor of mind and body* to *Preach the* GOSPEL of FREEDOM *throughout* the *U. S.* ONE GOD OVER and AROUND and IN ALL (*except a few devils*). I believe WOMAN an ANGEL of MERCY. But I forget these are but the '*illusions*' of a maniac (?) '*Insane* (?) from overworked brain.' My employers and Dr. Spray say this, on but limited knowledge of the *man* they are now *torturing* by *confining* with sons of GOD *de-*

*mented*," and so on through quires of letters and proclamations he formerly wrote. Lately he is too demented to attempt writing much.

Male case No. 301. Paretic Dementia. Frank E., age 45; admitted Oct. 4, 1883; German; married; clerk. Errabund; verbigerative. Was about to sue city for thousands of dollars damages. Said his wife was crazy; tried to have her arrested. Talked to angels who told him he would be rich (evidences of hallucinations of sight and hearing). Imagined himself worth millions of dollars; said he felt well; general anæsthesia decided; blind in one eye; varicosities in popliteal space; general tremor, with ataxic gait; drank hard formerly, but not lately. Had suffered from sun-stroke and head injury—bayonet-cut in head during the war, after which delirious two months; mornings usually more manageable; violent toward night, when delusions related to old army matters; thinks he is a prisoner of war surrounded by rebels. Sings vociferously "Battle Cry of Freedom" and other old patriotic songs. Grew rapidly worse; filthy, and furor occurred oftener. Died Dec. 18, 1883. Autopsy next day; skull thin and elastic abnormally; hæmatoma auris left ear. Blood in brain light-colored. No adhesions except intermeningeal along superior longitudinal sinus where membrane showed spots of extravasation. Vascular stasis apex right temporal lobe and basilar aspect, also in middle of left temporal lobe at base. Extravasations upper posterior aspect left cerebellum about a month old, forming bloody cyst in arachnoid half an inch in circumference. General vascular engorgement entire upper surface of cerebrum; puncta vasculosa numerous; ventricular endyma normal; cerebellum weight 6 ozs.; isthmus 1¼ oz.; left cerebrum 17 ozs.; right cerebrum 17¾ ozs. Total, 42 ounces.

Female case No. 165. Recurrent Mania. Ella A., age 43; Norwegian; widow; admitted May 5, 1882. Delusions of persecution, with optic and auditory hallucinations; furor every two or three months; father died of scarlatina at age of 33; mother died aged 72 years. When her menses began she was insane six months. A remission of five years followed, during which she married a drunkard who abused and neglected her. After birth of son, who is now 20 years of age, became insane again and was sent to Christiania Asylum, where she remained as a patient nine and a half years, and after remission of insanity was employed as an attendant. She and her son embarked for America in 1881, and the excitement of travel brought on third attack. In August, 1883, had an attack of typhoid fever in asylum, from which she convalesced by Dec. 15, 1883. During the height of this disease her furor appeared in a modified form, and the attendants could recognize the old incoherent ravings, but her weakness prevented their full expression. Jan. 1, 1884, noisy, destructive, singing, the past month; growing quieter till Feb. 2, 1884, when another outbreak of destructive fury appeared, but grew better daily, and is by the last of March quite rational, with a slight tinge of depression. She is very industrious during remissions, during which she talks rationally and realizes her condition. Is very fond of some of the ladies in the house, whom she recognizes and obeys, even in her greatest furor. May 3, 1884, is just recovering from a furibund attack.

Female case No. 168. Melancholia. Julia M., age 35; admitted May 17, 1882; Irish; married. Talks quietly but constantly on religious subjects to imaginary persons. Had been previously in poor health from nursing large child 10 months old. Parents died in middle life. Dec., 1883, improving rapidly; talks rationally; discharged recovered May 8, 1884.

Male case No. 97. Chronic Alcoholic Insanity. Martin H., age 40; admitted Feb. 18, 1882; Irish; married; night watchman; suicidal; delusions of persecution; says people tried to kill him, so he would kill himself, and save them the trouble; shot himself in breast; improved for some weeks after; sleeps restlessly; has large lupus erythematosus on nose, originated November, 1883; right malar region to left malar; ectropium induced by it in right eye; insanity accelerated by burglars having entered a store he watched. An ointment of hydrargyric nitrate, applied in March, 1884, rapidly changed the appearance of the lupus for the better. Erysipelas followed, abating in April.

Male case No. 331. Katatonia. R. H., age 24; admitted Dec. 13, 1883. American-German; single; salesman; had been gradually growing annoying, errabund, and eccentric for years, appearing to tend toward hebephrenia; committed many improprieties; last summer much worse, and drank hard. Jan. 9, 1884, hysteroidal convulsions followed stagy behavior; then began cataleptoidal stage, during which his pulse was feeble; temperature subnormal; lay abed with eyes rolled up, but semi-conscious; winks, and has waxy mobility. Feb. 4, more difficult to rouse; some oozing of saliva from corners of mouth. Feb. 7, gradually emerged from stupor, and broke a window; maniacal stage began Feb. 8; cannot be kept in bed; sleepless. Feb. 14, quieter; remains abed; eats and sleeps well. Feb. 16, still violent; strikes patients. Feb. 18, quieter, Feb. 20, emaciated; stupid; talks incoherently; noisy at night. Feb. 25, fever began. March 3, quarrelsome; sleeps well, and is growing fat (of bad prognosis). March 15, attempted to strike superintendent, and uses vile language. Excitement gradually merging into sullen melancholia by May, 1884.

Male case No. 332. Primary Confusional Insanity. Wm. I. M., age 25; admitted Dec. 20, 1883; American; single; salesman. In Sept., 1880, was in Oakland, California, Asylum for awhile after a spree. Upon this occasion exhibited peculiarities of primary confusional insanity rather than those of acute alcoholic, although the cause was a protracted debauch. Restless; incoherent; talked of betting on the white horse; would repeat questions asked him; talked of headache occasionally, and rambled something as follows: "White horse; there he is; didn't get there; get up; hello; yesterday; bet your life; where did he go; he has a bad eye; look out; let's take a drink." Jan. 7, recovered his mind suddenly. The last he remembers is being in the Union Depot, and when he became sane thought he was still there, and began to wonder at people sitting around with hats off and some without coats. The time he was insane is completely blotted from his memory. Sent to his father in Iowa in custody of friends Feb. 4, 1884.

Male case No. 348. Melancholia. M. T., age 29; admitted Jan. 31, 1884. Italian; single; statuary; delusions of persecution; suicidal; had been working in terra cotta factory exposed to great heat. In the same room here with him was male case No. 123, from whom he soon imbibed the notion that he too was to be hanged, affording the *folie a deux*, or communicated delusion, to which French alienists first called attention. This sometimes runs through a family. In 1879, three insane sisters agreed to hang themselves, having identical delusions of persecution, and two of them succeeded in committing suicide at their residence on Langley avenue near 39th street, in Chicago. There was an undoubted teratological defect in these sisters. *Folie a deux* is not uncommon ; nearly every asylum affords one or more cases.

Female case No. 180. Melancholia. Kate G., age 25; Irish ; domestic ; married ; suicidal ; June 3, 1883, escaped vigilance, and sprang through elevator door, falling four stories, comminuting left tibia four inches and dislocating both ankles. Dec. 31, 1883 ; confined to bed ; recovering from injuries slowly ; talks rationally, except in persisting in her suicidal desires ; says it is only a question of time when she will succeed in killing herself.

Female case No. 181. Hebephrenia. Theresa S., age 20 ; German ; domestic ; single. Noisy, destructive, untidy ; laughs sillily at times, incoherent ; said to have become insane when menses stopped. Under treatment the flow was reëstablished, but no improvement in mind occurred.

Female case No. 182. Mania. Ann D., age 40 ; Canadian ; seamstress. Admitted July 27, 1882. Fancies that she is wealthy, and that her sister cheats her. Given to lying about everyone. Delusions of persecution. Verbigerative, incoherent, harmless ; scolds ; not destructive ; lazy, but tidy. Father died through accident when aged 60 ; mother failed in health after climacteric, and died aged 58. Patient's menses stopped three years before admission, and she began to be suspicious and melancholy, followed by persecutory delusions and fear of poisoning.

Female case No. 176. Epileptic insanity. Nellie McC., age 30 ; American ; married. Admitted June 29, 1882. Between paroxysms works in sewing room. What is known as the psychic equivalent appears in her case, for example, she often has furor lasting two days and nights, during which she fights. Dec. 30, 1883, fell to floor ; mouth twisted to one side the only convulsive movements noticeable; became silly afterwards for a few hours, but went to work as usual, and mind cleared up till Feb. 7, 1884, when an outbreak of fury occurred. The furor taking the place ot the epilepsy constitutes the equivalence, or occasional larvated form of that disease. She has four children, the youngest born in 1880. Husband first became aware of her insanity when, one morning, he was awakened by her beating him in the face with a slipper. She is wholly unconscious of these acts, and insists upon there being a mistake. Her demeanor between attacks is quite gentle. Naturally industrious.

Female case No. 170. Melancholia. Ida L., age 32; German Jewess; single. Always apprehensive that something terrible is about to occur. In 1866 was in Hartford, Conn., asylum two years; 1870 two years more in same asylum. Her sister committed suicide a year ago over a love affair. When Ida's insanity re-appeared she tried to drown an infant sister in a tub, explaining it was better they should all die. Says she cannot understand how any one could wish to live. Talks rationally, and has ladylike manners. Talks and sings in her sleep so loudly she has to be often awakened by night watch. Feb. 8, 1884, transferred to private asylum.

Female case No. 169. Mania. Paulina H., age 34; Swede; married. Hallucinations of hearing; thinks the sun and stars dictate to her; quiet usually, and talks coherently; has been neuralgic; flushes easily if spoken to suddenly. Opium and alcohol addiction alleged causes of her insanity.

Female case No. 166. Mania. Annie M., age 38; Irish; single; admitted May 11, 1882. Seven years insane; hallucinations of sight; sees dead relatives; quiet; talks on religious subjects incoherently if addressed; furor about every third month, when she throws things about, but quiets quickly. Amenorrhœa, costiveness, and dysmenorrhœa. Father died of cholera, and mother of some form of heart-disease. Patient was always self-willed. A cousin was insane after childbirth, and recovered after a barn adjoining house had burned. Annie has suffered from painful menstruation since 18 years of age, when catamenia began.

Female case No. 105. Mania. T. P., age 33; German; married; admitted Jan. 28, 1880. Frequent furor, lately somewhat more peaceable; extremely jealous of her husband; accuses nearly every female of trying to take him from her; was weakly on admission, but health improved later; has dysmenorrhœa and scanty menses. Sister in Jacksonville asylum incurable, exciting cause in that case alleged to be death of children.

Female case No. 103. Terminal Dementia. Florence B., age 27; American; married; admitted Sept. 17, 1879. Usually sits bent forward, with head resting in right hand and eyes closed, occasionally starts up with incoherent exclamation. At the entertainments given in the asylum, she is noticeable as an excellent dancer, always looks neat, but this is more owing to attendants' than patient's care. Before and after menses, has monthly furor, during which she is stubborn and fights, but not noisy. Insanity said to be hereditary. She had six children in seven years, with other unusual domestic trouble .

Some Continental and English asylums would classify this case among chronic puerperal, but Krafft-Ebing has indicated the inappropriateness of so doing. Puerperal cases either recover or pass to secondary confusional, and then terminal dementia, as do the other psychoses.

Female case No. 88. Secondary Confusional Insanity. Julia H., age 46; Irish; married; admitted Oct. 10, 1870. Noisy, constantly prays, sings, and blesses everyone; sometimes belligerent. Her mother was confined after a fright at seventh month of gestation when Julia was born. General health, never very good. She is the mother of nine children, one of whom suffers from coxalgia; since youngest child was born began to "act strangely," constantly going to church and remaining till expelled; depressing delusions then began; attempted suicide by strangulation. Says that drunkards are possessed by the devil, which is about the only fixed idea to which she clings.

Female case No. 52. Agnes S., age 51; Bohemian; married; admitted March 30, 1876; early history not obtained; was insomnolent, with occasional attacks of dizziness and slight frenzy; usually very pleasant and rational; pulsation of left carotid artery visible from twenty feet away; cervical vessels generally dilated; suffered intensely toward close of her life; she died at midnight of August 12, 1883. Autopsy next day revealed liver enlarged and congested; lungs emphysematous and melanotic. Hypertrophied heart measured 14 inches in circumference at auriculo-ventricular junction, 7 inches from apex to base; outer part fatty; weight with 2 inches of aorta, 24 ounces; aorta full of calcareous plates; arterial atheroma general.

Her physical condition rendered hospital treatment necessary, but no especial psychosis need be erected to fit her case, beyond recognizing the occasional delirium.

Female case No. 30. Secondary Confusional Insanity. Jeanette W., age 24; American; actress; single; admitted March 8, 1873; went by stage name of Capitola Delzell for some years until real name ascertained; she is over usual female height, with a stately figure, and evidently was a beautiful woman. During the day she engages in light housework, has little to say to anyone, is trusted to go about the building, attends to what she is directed to do quite well, but her ideas are otherwise foggy, and memory impaired. She can communicate her desires readily enough in short sentences, but from the blank stare of her eyes and occasional mutterings and incoherent addresses to invisibles, she is doubtless hallucinated. Rather reticent by day, but about two nights every week she is furious, swears, scolds and hammers on her room door, declaring that some one tries to break into her room and that her side is cut open.

Male case No. 20. Secondary Confusional Insanity. Charles E., age 27; admitted Jan. 11, 1873; American; printer; Protestant; single. Became insane at puberty with the usual silliness and occasional destructiveness of hebephrenia, many of the peculiarities of which he still preserves. Destroys his clothing, chews scraps of paper and rags, mimics others, and has very clownish manners; constantly in motion, but not belligerent; is mischievous to an extreme. Has three brothers insane, and it is reported that other members of his family are also insane, also that only one of the family living is sane, a sister, who visited him in Nov., 1882. He recognized her, and talked of old times, wept, and showed more evidences of latent rationality, than ever before or since, while here. Sister said patient was in the Union Army, and suddenly became insane at battle of Vicksburg. His father, who was at one time the Governor of an adjoining State, died suddenly after severe exertion, supposed apoplexy. Mother died from some form of heart disease. Patient has sleepy, stupid periods monthly, lasting about a week.

Male case No. 40. Katatonia. Adolph C., age 28; admitted Feb. 25, 1877; German; Hebrew; single; tanner. Slight catalepsy merged into stupor once in 3 months, comes on suddenly over night, recovers from it slowly, then fairly intelligent and pleasant; furor appears suddenly, lasting a week or ten days. Last furibund attack Feb. 7, 1884.

Male case No. 42. Epileptic Insanity. Charles C.; admitted April 26, 1877; age 20; Irish; Protestant; single; patternmaker. Insane eight months before admission; beginning of epilepsy unknown; exhibits mental hebetude; never attempted mischief; is unconscious a little while after paroxysms, which appear daily, often three or four attacks in twenty-four hours.

In many cases of insanity complicated with epilepsy, after terminal dementia has been reached, as in this case, the epileptic paroxysms cease altogether. In this instance the dementia is not profound, but the epilepsy persists. The varying phases of this form of insanity are well worthy of deep study, as offering a probable solution of much that is now unknown in cerebro-pathology and epilepsy. Any asylum official who will carefully record all he can gather relating to his epileptics, will be able to make some valuable deductions in time.

Male case No. 48. Chronic Alcoholic Insanity. John A., age 22; admitted March 12, 1870. Had been on a protracted debauch before admission. Is now stupid, amnesic, inclined to mutism; sleeps fairly well; tongue tremulous; pulse quick and compressible; heart's action violent; fairly nourished; complains of sickness in stomach and head; tries to escape; troublesome. Has idle, obstinate periods about once or twice yearly, when he refuses to work. Is confused, erotic, and has hypochondriacal delusions; usually works on the farm.

Male case No. 57. Secondary Confusional Insanity. Mortimore McC., age 50; admitted Nov. 1, 1878; Irish; married; laborer. Loquacious; pedantic; self-complacent; dresses neatly; says he owns $4,000 property in Vermont, imagines himself a great admiral and talks much of naval fights; was two years in Elgin asylum before admission here. Had delusions of persecution and grandeur at home. Threatened daughter, but otherwise not homicidal or suicidal. Did not drink to excess; was struck on head when 20 years of age while trying to defend a woman from abusive husband. Injury apparent over posterior fontanelle; depression of bones, teeth defective. Had typhoid fever when a young man. Since Dec., 1884, more excitable, increasing by March, 1884. Talks incoherently until questioned, and then answers connectedly.

Male case No. 59. Secondary Confusional Insanity. Daniel P., aged 32; admitted Jan. 26, 1879; American; married; bricklayer. Was in Andersonville prison during war, since when has complained of pain in chest, left side; two years afterwards began dizziness with night sweats, congestive attacks with cramps followed, for which he was treated with cod-liver oil. Mental trouble began with delusions of persecution five months before admission. Until Dec. 30, 1883, worked in asylum kitchen, but tried to stab a fellow-patient and was, since then, kept on ward. No heredity. Delusions of grandeur. Imagined he knew more than any one in the world, owned everything, and could scoop up diamonds from his window sill. The present phases of his insanity indicate that paranœa was his primary defect.

Female case No. 162. Hysterical Insanity. Fanny F., age 21; admitted April 4, 1882; German; Hebrew; single. Incoherent, hysterical, lies on floor, generally sillier every other day; weakly. Irregular and painful menstruation. Aunt insane in this asylum.

Female case No. 153. Mania. Elizabeth G., age 50; admitted Jan. 14, 1882; German; married. Insane two months before admission while convalescing from attack of variola. Has a brother insane in Germany. Imagines she is rich and has other expansive delusions; harmless; no persecutory delusions; sighs and talks incoherently at times, generally quiet, neat; works on ward, keeps her head bound around with handkerchief, for, she says, her head aches. Last attack of furor Feb. 21, 1884.

Female case No. 151. Terminal Dementia. Maggie W., age 39; Pole; single; domestic. Last admission Dec. 1, 1881, previously here three times since 1877. Began as melancholia with delusions of persecution, and sometimes hallucinations of sight. Complained much of headache. Mother died of "dropsy." Patient had been working hard and caught a severe cold. Nostalgia and her mother's death preyed upon her mind greatly.

Female case No. 149. Epileptic Insanity. Belle G., age 14; admitted Oct. 20, 1881; American Celtic; single. Epileptic every two or four months since 5 years of age. Talkative before or after paroxysms, usually before. Between attacks grows haggard and old-looking; reticent. Once for 24 hours last year, spit froth all over her room continuously. Epilepsy said to have originated through a fright. Is rendered stupid and more childish after each paroxysm. Feb. 7, 1884, epilepsy. Feb. 10, silly, obtrusive; twists face to left side in talking. Feb. 16, succession of convulsions past three days. Feb. 27, one attack. Recurrences often in March and April. May, 1884, more demented.

Female case No. 148. Secondary Confusional Insanity. Ida M., age 36; admitted Sept. 29, 1881; German; married. Four children, youngest 7 years old, after birth of which she became insane, hence originally puerperal insanity, which has advanced to the terminal condition of secondary confusional. Was at first extremely furibund and dangerous, but began with raptus melancholicus (the terrorized fury of melancholia), sitophobic, with other delusions of persecution. Formerly at Elgin asylum, and has had a previous attack. Heredity denied, but her mother died of nostalgia, which does not support the claim. Patient sews; cries occasionally because detained here. Scolds about domestic affairs, pins papers to her hair, and otherwise shows progress toward terminal dementia.

Female case No. 133. Secondary Confusional Insanity. Louise H., age 44; admitted March 31, 1881; German; domestic; widow. Imagines she is rich at times. Incoherent; cheerful usually, but scolds if opposed and talks vilely; idle; was in poor health upon admission. Mother died aged 73, with "enlarged liver." Father living and healthy. Patient had previous attacks, two or three in last 15 years, beginning with "stomach cramps," following business trouble. Lost property through law-suit. Had eleven children, one of whom at least is advanced in phthisis pulmonalis. Her insanity began with violent furor.

Female case No. 132. Hypomoria. Maria B. N., age 55; admitted March 24, 1881; Alsatian; married; strong and stout. "Father slightly insane after a law-suit with brother." Great chatterer and scolder. Has a very pleasant voice and sings much, but scolds more, not angrily, but as though jesting. Fond of playing pranks upon her fellow-patients. Is very jolly, but tiresome withal, for her exuberance lasts from morning to night, and from one year's end to the other. Delights in being entrusted with keeping order upon the ward, and is an efficient worker.

Female case No. 131. Secondary Confusional from mania in puerpero. Mary C., age 29; admitted Feb. 6, 1881; Irish; married. Grandmother died in insane asylum in Ireland. Insanity began with depression when child was four months old, but rose to furor soon afterward. Has grandiose delusions; talks to visible and invisible people about the wealth and respectability of her people; maintains a haughty bearing, and makes faces at all around her. Irritable always, and obtrusive, but not destructive or otherwise offensive.

Female case No. 115. Mania. Hannah B., age 37; admitted Aug. 25, 1880; Irish; married. Verbigerative; incoherent; sleepless; delusions of persecution; talks vilely when furibund; at other times is fairly coherent. Says when 13 years old was struck on head with a skillet, and three days after became insane, and was taken to an asylum in Ireland. Verdict states her insanity as hereditary, but does not mention source of information.

Female case No. 114. Terminal Dementia. Eunice S., age 50; admitted June 17, 1880; American Celtic; married. Sleepless; neat habits; imagines her husband is coming for her daily; amnesic; cannot tell people apart; no furor, but objects and squabbles when being bathed. Domestic trouble alleged cause of insanity, which began as melancholia. Her menses were irregular six years before admission, and about the time of appearance of melancholia, she expectorated blood for a day each month.

Male case No 67. Paretic Dementia. Joseph Le V., age 33; admitted Feb. 5, 1880. Pupils irregularly dilated; speech and gait ataxic; general anæmia. Verdict alleges a hereditary cause of insanity. Was filthy and covered with vermin upon admission. Ataxia increased by March 5, 1880, and by last of September, 1880, was quite demented, in which condition he has remained latterly, confined to bed.

Male case No. 65. Secondary Confusional Insanity. Wm. B. D., age 22; admitted Oct. 9, 1879; American; clerk; single. No heredity; refused to speak; was bill clerk in a large wholesale liquor store in Chicago; drank to excess; discharged and taken to Utica, N. Y., Insane Asylum. Had syphilis in 1875. During 1880 worked a little on ward. March 1, 1881, filthy, eating his own excrement; decorates room with filth. Cacophonous; yells constantly "king crown, king three crown, mealy moe." Crosses his hands when he wishes to shake hands with any one. Not violent unless thwarted.

Male case No. 71. Epileptic Insanity. Daniel D., age 31; admitted Nov. 28, 1880. Very violent at first, less so latterly; head injured when 7 years old; left parietal region depressed. Recent epileptic seizures Jan. 1, 1884; Feb. 13, with furor following at night; Feb. 27, fell from bed in convulsion; Feb. 28, another attack, and one upon March 15, 1884.

Male case No. 86. Secondary Confusional Insanity. Jas. D., age 42; admitted July 28, 1881; Irish; married. Seven years insane before admission; expansive delusions; quiet; fixed delusions of grandeur; thinks he is a great doctor and very wealthy; formerly furious; quiet now; thinks free-masons are after him. Was sunstruck, and sustained an injury to head in addition. Mother died of phthisis pulmonalis.

Male case No. 84. Epileptic Insanity. August S., age 24; admitted April 22, 1881; German; single. Five to ten epileptic seizures daily, with a remission of two weeks or thereabouts. Irritable after fits; threatens suicide; attacked brother-in-law; works during remissions. Condition after attacks varies, often may be demented or irritable. Feb. 18, 1884, attack, followed by severe vomiting; Feb. 24, 1884, violent furor after fit; tried to strike and bite attendant 48 hours afterward.

Male case No. 74. Mania. Hubbard S., age 25; admitted Dec. 16, 1880; laborer; single. Was a convict in Joliet prison Feb. 2, 1870, and three years thereafter became insane. Unsystematized delusions, frequent furor, fights and tries to escape. Pyromaniacal. He set fire to this building three times before caught at it. Verdict states his imprisonment as cause of insanity, and this suggests the medico-legal points often involved in such cases. Paretics often commit the fights and other crimes in the beginning of their insanity, are sent to prison—the mental failure at this time not being easy to recognize—and when the insanity becomes gross enough to be noted by the prison physician, they are rejailed in asylums as rendered insane "through imprisonment." Requisite knowledge of this disease at the outset would save many a family the odium of criminality alleged, where there was pure insanity instead. The history of this particular case is not well enough known to enable the statement that this patient was or was not insane before imprisonment. But, while the insane are often sent to the penitentiary and hanged for crime (recently a madman was carried to the gallows in New Jersey in a strait jacket), the contract system of leasing labor in penitentiaries causes insanity direct. On the Mooney trial, in this State, Dr. Kiernan informs me, it was testified that twenty-five lunatics have been sent from the wire department of the Joliet prison within the recollection of one man. Dr. K. says the narrations on that trial sounded like those in Charles Reade's "Never Too Late to Mend."

Male case No. 88. Secondary Confusional Insanity. Edward C., aged 24; admitted Dec. 1, 1881; American Celtic; single. Arrested in Chicago and convicted for being principal in robbery and red pepper throwing operation with a negro and boy, wherein a large amount of money was taken. Was always bad, unreliable and treacherous; kicks, bites, strikes, and spits at every one; mutters in sleep. Father myopic and mentally "peculiar," mother sane; sister blind; brother myopic. Rapidly passing to terminal dementia.

Male case No. 98. Paranœa. Daniel O'C., age 25; admitted Feb. 27, 1882; Irish; single; laborer. Scorbutic December, 1883; fancies he was mistaken for Guiteau; says he will not leave the asylum till he has damages for false imprisonment. Usually quiet, but when excited talks of Guiteau trial. Says there is no law in the land to protect greenhorns like himself. Makes stump speeches favoring land-leaguers, and against the English.

Male case No. 99. Epileptic Insanity. John S., age 18; admitted March 1, 1883; American Celtic; single. Father died with phthisis pulmonalis. Patient, when 6 years of age, had severe pneumonia, followed by epilepsy, five to ten fits daily, with remissions of several weeks. After paroxysms very morose, and easily angered, then delusional and destructive; broke three doors and several windows once at home; threatened to kill step-father, whom he always disliked. During remissions is quiet and rational. Had a severe fall some years ago upon head, which confined him to bed awhile.

Male case No. 100. Secondary Confusional Insanity. John McN., age 36; admitted April 13, 1882; Irish; single; blacksmith. Suicidal; refused food; prayed loudly at first; talks coherently; usually quiet; refuses to work; gesticulates; has delusions of persecution; thought people would kill him. Hallucinations of sight and hearing. Saw and heard the dead walk, and talk. Was intemperate and religiously excited. Cousin on paternal side committed suicide while insane.

Male case No. 102. Epileptic Insanity. George M., age 22; admitted March 30, 1882; American; single; laborer. Sometimes has epilepsy, and no outbreak follows, but is very destructive when it does follow. Often has full "warning;" then asks attendant to take care of him, as he is about to have a fit. Has remissions of a month, but the least excitement, as a dance or dispute, causes them to recur. Ran away several times; once engaged to a farmer, who did not discover his malady for some time. Broke down door here once. Last attack March 10, 1884, with great furor following.

Male case No. 115. Chronic Alcoholic Insanity. Christian L., age 58; admitted July 13, 1882; German; married; farmer. Eye injured; says it was scalded out. Very intemperate. Tries to kill his wife every time he returns home. Talks rationally, but full of delusions concerning his wife.

A remarkable feature in alcoholic insanity is the usual delusion of marital infidelity. Wherever this delusion appears, the alcoholic causation may be safely presumed.

Male case No. 124. Chronic Alcoholic Insanity. E. R., age 62; admitted August 24, 1882; German; married; musician. Drank excessively, and became abusive and dangerous to relatives. Was leader of a musical society for ten years in Chicago, and a recognized proficient in music. Amnesia of senility; cannot remember recent events from one day to another, but remains an excellent musician. Has difficulty in recalling names of his pieces, but plays them when the tune is started for him. Has the delusions pertaining to marital matters common in inebriates advanced to this stage, and cannot be kept at home owing to his abusiveness to wife and children. At all times insists that he has been here only four or five weeks. Delights in teaching music and playing for amusement of patients. Is quite a favorite with everyone here, and it is difficult to realize how great his mental failure is, while his every day demeanor here is irreproachable. It is commonly observed that drunkards who may be demons abroad, exhibit latent excellencies of character during asylum sojourns which were little suspected before as existing at all in them.

Male case No. 135. Epileptic Insanity. Samuel A., age 32; admitted September 21, 1882; American; single; machinist. Pyromaniacal and suicidal. Often six to ten epileptic attacks nightly, followed by sullenness and violent furor. Has delusions of persecution and hallucinations. Liquor or tobacco injures him, by rendering epilepsy more frequent. Has been epileptic nine years. Attacks approaching indicated by his singing and noisiness; makes peculiar opisthotonic movements generally before fits. February 14, 1884, post and pre-epileptic furor; struck a patient with spittoon, upset tables and benches, and fought attendants. February 16, furor as before; broke window during night. March 3 and 18, 1884, furor followed epilepsy.

Male case No. 140. Mania. Jos. H., age 22; admitted October 19, 1882; single; carpenter. Has subjective sensations of having been struck, as he frequently whirls around and strikes others, claiming he was struck by them first. Complains of pains in knee mornings; says he is rheumatic, and that his chest hurts him. During furor, bit an attendant's leg severely. Improving since December, 1883.

Male case No. 142. Secondary Confusional Insanity. John R., age 27; admitted October 26, 1882; German; single; maltster. Insanity began as mania one week before admission. Imagined the devil was after him; suicidal; incoherent; talked incessantly; quarrelsome when disturbed; hallucinations; complains that people accuse him of murder and arson, and that he is guiltless of either. Corpulent. Mother, grandmother, and sister insane.

Male case No. 144. Choreic Insanity. Frank G., age 20; admitted November 2, 1882; American; single; upholsterer. Chorea began four years before admission, gradually merging into insanity. Obstinate; costive; noisy at night; mischievous; destructive; talks foolishly. Father died of "paralysis." Patient was greatly benefited, physically, by Squibbs' fl. ext. conium, and was tried at home, but returned in four days, December, 1883. Is in constant motion, jerking body in all directions. Gradually growing demented.

Male case No. 307. Melancholia. Anton R., age 25; admitted October 25, 1883; Pole; married; gilder. Was afraid of everyone; thought doctors after him to cut him up. Liquor in small quantities affected him badly; suffered from headache. Stole $170 from a saloon-keeper, and afterwards returned $70 to police voluntarily. Mother also in asylum, female case No. 368. Some indications of plumbic toxæmia led to appropriate treatment, and he grew better mentally awhile, but relapsed.

Male case 154. Melancholia. Alex. H., age 42; admitted Dec. 8, 1882. American; married; railroad fireman. Delusions of persecution. Insanity began in 1879, when his youngest child was run over by cars and lost an arm. He was never intemperate. All his relatives nervous and excitable. His father died in arrisburg, Pennsylvania, Asylum, in 1840; sister died in Jacksonville, Illinois, Asylum, in 1872.

Male case No. 161. Secondary Confusional Insanity. Wm. H., age 44; admitted Dec. 21, 1882; American; laborer; single. Preserves much of former maniacal disposition. Incessant, incoherent, vile talker; was in Stockton, California, Asylum. Constantly talks of A. G. Shurtleff, the superintendent of that place. Is the vilest-spoken male patient here, and not to be trusted with his fists, but is allowed occasionally to take a run outside without the least danger of his leaving. It is surprising to notice how much will power he can exert. At the Saturday evening dances he behaves himself, utters not a word, and evidently restrains himself by keeping his lower lip clenched between his teeth to prevent the usual torrent of abuse escaping him. His passage toward terminal dementia is slow but noticeable.

Male case No. 167. Senile Dementia. E. W. W., age 73; admitted Jan. 27, 1883. American; married; farmer. Usually mild-mannered and quiet; feeble with age. Occasionally hallucinated and incoherent. Assistant physician Thuemmler noted that his attacks of mild furor are preceded by patient's sticking his pants in his boots, and the doctor finds this an excellent signal for treatment.

Male case No. 173. Melancholia. Anton D., age 55; admitted Feb. 15, 1883; German; married; expressman. Poorly nourished. Relatives state that in 1879, burglars chloroformed him, and he never was the same man afterward. Tried many ways of self-destruction, even once by starvation. Delusions of persecution; thinks every one wishes to kill him; refuses to accept anything from his relatives, as he thinks they are starving.

Male case No. 197. Melancholia. John S., age 58; admitted April 12, 1883; English; married; clerk. Temperate. Sat up nights to watch his children, fearing something would befall them while they slept. Was wealthy at one time, and while actively employed, thought little of his misfortunes. Four months before his admission, lost situation, and melancholia followed; thought his food was poisoned. Tonics, laxatives, and at night sleeping potion administered; gradually recovered, and discharged August 7, 1883.

Male case No. 132. Mania. C. K., age 43; admitted June 7, 1883; German; married; commerce; insane one month before admission. Thought he saw ships and sailors, drew marine sketches; mind reverted to his sailor days. Was overworked and had business trouble; had always been kind to wife before, but turned against her while insane; struck her in face in December, 1883, during a visit she paid him. February, 1884, began to recover, and March 17, 1884, sent home apparently recovered.

Male case No. 242. Mania. Fred. P., age 60; admitted June 28, 1883; German; married; tailor. Was arrested in 1870 for some trivial matter, and became maniacal in jail. Had been intemperate; tried to set fire to his son and to kill his other two sons. Furor recurred about monthly, lasting one or two days. During remissions pleasant and jocose, but easily excited. Ran in streets when furibund. People made fun of him, and saloon-keepers dosed him with swill beer and filth. Died July 6, 1883. No autopsy allowed.

Male case No. 272. Acute Alcoholic Insanity. Wm. H., age 40; admitted Aug. 9, 1883; Norse; widower; cooper. The cooper for whom he worked noticed his face flushed and that he appeared confused. Said he was waiting for a man; trembled violently. Discharged recovered Sept. 16, 1883.

Male case No. 277. Mania. James F., age 25; admitted Aug. 16, 1883; American; married; laborer. Insanity began October, 1882. Over-mental strain alleged cause. Delusions of grandeur and persecution; hallucinations of vision and hearing; very jealous of wife. Furor every two or three weeks; head cut by bruisers right parietal region and over top of frontal region. Father irritable; mother insane since 1878; sister in Wisconsin Insane Asylum. Wrote a letter to Gen. Phil Sheridan, asking him to take him hence.

Male case No. 224. Paranœa. Christian S., age 50; admitted May 31, 1883; German; farmer; quiet and industrious. It required considerable questioning to bring out his delusions. He thinks a certain neighbor bewitched him, and it is necessary for him to fire off a gun once in a while to break the spell cast over him. His otherwise apparent sanity led to the issue of a *habeas corpus*. In court Dr. Thuemmler was asked why the patient had not been discharged from the asylum. The doctor replied, because the medical men did not wish to take the responsibility of turning loose a monomaniac, who might do harm, When asked if he thought the patient's delusion might lead him to shoot his neighbor, Dr. T. said he could tell as little about that as the judge could, and that as to allowing him his liberty, it was for the judge to decide the advisability of so doing. The superintendent of the asylum was not, under the circumstances, willing to assume the risk of such discharge. He was remanded to the asylum. He thinks forty-two different witches crawl down his throat, and that ghosts talk to him. Upper part of nose deeply indented by kick from a horse when patient was a boy. Frontal injuries, in my observation, are frequently associated with paranœa.

Male case No. 69. Paranœa. Anton N., age 49; admitted July 8, 1880; Poland; cabinet-maker. Is very ingenious at his trade; has made for himself a full set of tools used in his occupation from odd pieces of wood, iron, glass, tin, etc.; makes very tasty cabinet work of all kinds. I have a very handsome frame made by him to hold panes of glass as a cover for my laboratory microscope. The joinery is perfect. When this patient is "off," he yells defiance at the devil. He cut a dragon-winged image of a female from wood and suspended in his window to bother Lucifer "with a picture of his wife." I asked him once if he would not like to be at liberty. "Yes," he replied, "I would be happy if I could go to Chicago to kill two men there who are in league with the devil against me." Noticing the facial anæmia during his paroxysms, they can usually be averted by stimulants.

Male case No. 212. Paranœa. Gottlieb H., age 40; admitted May 10, 1883; German; painter; single. Imagines temperance people and Lutheran priests persecute and follow him; well-behaved and good-natured; assistant to asylum painter; verbigerative when talked to; anæmic at times; complains of occasional headaches; frontal transverse rugæ well marked, particularly when talking. He says both his parents are in an insane asylum in Eslingen, Germany. He carries scraps of newspapers detailing an attack he made upon a Mr. C. H. Peck, of Atchison, Kansas, in 1880. Says he was struck by lightning in September, 1880, but was not hurt; felt only a little burning sensation in his stomach. A photograph of himself, which he says was taken in January, 1883, resembles him but little now. The picture shows a man of ordinarily intelligent appearance. He must have deteriorated in features rapidly. Has transitory hallucinations, and desire to get justice for having been sent here. Answers description of querulous monomania, "*Querulanten-Wahnsinn*" of the Germans.

Male case No. 289. Melancholia. J. S., age 30; admitted Sept. 13, 1883; German; married; printer. Sensational case published in Chicago *Tribune* Sept. 14, 1883. Fell in love with a young girl who lived with his wife. The feeling seems to have been reciprocated, for the young lady committed suicide, and both patient and his wife identified her body when taken from the lake. Later, patient attempted suicide in river. He had unsystematized delusions of persecution, with hallucinations of sight; thought he saw the young lady alive, and also many of his friends who had long been dead. His mind became clouded at the inquest of the young girl, and he walked about with a dazed, troubled expression. It was with difficulty he could be led to talk of anything but his misfortunes. Anæmia indicated tonics, and much was done to interest him in general matters. He was finally induced to read articles in *Zeitschrift für Psychiatrie* and other German medical journals, and seemed startled into recognizing cases similar to his own reported therein. By the last of September his mind had become decidedly clearer, and Oct. 14, 1883, he was discharged. Jan. 1, 1884, he called to convince us of his recovery. He is a compositor on a German paper. Says he is seldom depressed now, and when ideas of persecution arise he reasons himself out of them, recalling the instruction he received while here. The possession of a mental background which may be educated to withstand insane tendencies is an all-important feature in some forms of insanity; of course, in paretic dementia it avails nothing, but should be appealed to wherever hope exists.

